# Multivariate Pattern Classification of Primary Insomnia Using Three Types of Functional Connectivity Features

**DOI:** 10.3389/fneur.2019.01037

**Published:** 2019-10-02

**Authors:** Chao Li, Yuanqi Mai, Mengshi Dong, Yi Yin, Kelei Hua, Shishun Fu, Yunfan Wu, Guihua Jiang

**Affiliations:** ^1^Department of Medical Imaging, Guangdong Second Provincial General Hospital, Guangzhou, China; ^2^Maoming People's Hospital, Guangdong, China; ^3^Department of Radiology, The First Affiliated Hospital of China Medical University, Shenyang, China; ^4^Department of Medical Imaging, The Affiliated Guangdong Second Provincial General Hospital of Southern Medical University, Guangzhou, China

**Keywords:** primary insomnia, insular cortex, frontal lobe, machine learning, support vector machine

## Abstract

**Objective:** To explore whether or not functional connectivity (FC) could be used as a potential biomarker for classification of primary insomnia (PI) at the individual level by using multivariate pattern analysis (MVPA).

**Methods:** Thirty-eight drug-naive patients with PI, and 44 healthy controls (HC) underwent resting-state functional MR imaging. Voxel-wise functional connectivity strength (FCS), large-scale functional connectivity (large-scale FC) and regional homogeneity (ReHo) were calculated for each participant. We used support vector machine (SVM) with the three types of metrics as features separately to classify patients from healthy controls. Then we evaluated its classification performances. Finally, FC metrics with significant high classification performance were compared between the two groups and were correlated with clinical characteristics, i.e., Insomnia Severity Index (ISI), Pittsburgh Sleep Quality Index (PSQI), Self-rating Anxiety Scale (SAS), Self-rating Depression Scale (SDS) in the patients' group.

**Results:** The best classifier could reach up to an accuracy of 81.5%, with a sensitivity of 84.9%, specificity of 79.1%, and area under the receiver operating characteristic curve (AUC) of 83.0% (all *P* < 0.001). Right anterior insular cortex (BA48), left precuneus (BA7), and left middle frontal gyrus (BA8) showed high classification weights. In addition, the right anterior insular cortex (BA48) and left middle frontal gyrus (BA8) were the overlapping regions between MVPA and group comparison. Correlation analysis showed that FCS in left middle frontal gyrus and head of right caudate nucleus were correlated with PSQI and SDS, respectively.

**Conclusion:** The current study suggests abnormal FCS in right anterior insular cortex (BA48) and left middle frontal gyrus (BA8) might serve as a potential neuromarkers for PI.

## Introduction

Primary insomnia (PI) is the most common sleep disorder and is a major risk factor for depression, and in certain instances could increase mortality (1). At present, diagnosis for insomnia is mainly based on self-reported sleep difficulties. Objective neurobiological markers remain largely unclear and hence prevented the development of more cost-effective, efficient, and accessible therapies (2).

Non-invasive neuroimaging technology opens a window for the study of neuropsychiatric diseases, include insomnia (3–5). Previous studies found aberrant brain metabolism and connectivity related to the prefrontal cortex, insular cortex, amygdala, precuneus, and caudate in primary insomnia (5–18). For example, using PET, Nofzinger et al. (6) found a smaller decrease in relative metabolism from waking to non-REM sleep states in the ascending reticular activating system, hypothalamus, thalamus, insular cortex, amygdala, hippocampus, anterior cingulate, and medial prefrontal cortices, which supports the CNS hyperarousal hypothesis. Using independent component analysis, Chen et al. (9) demonstrated that the anterior insular cortex had greater involvement with the salience network in PI. This greater involvement was also correlated with self-reported alertness and negative affect. This study highlights the importance of the salience network in hyperarousal and affective symptoms in insomnia. Stoffers et al. (15) found that hyper-arousal was associated with reduced caudate recruitment when performing an executive task. Interestingly, attenuated caudate recruitment did not recover after successful treatment, suggesting abnormal caudate activation is a potential vulnerability biomarker for insomnia. Recently, Lee et al. (8) observed that subcortical FC was changed after cognitive–behavioral therapy, which suggested that FC may be a biomarker for tracking response to treatment.

While these studies were valuable in finding relevant neuroimaging biomarkers, the studies were based on group comparisons, and hence was not sufficient for possible translational applications, such as for direct clinical diagnostic and prognostic evaluation (19). Up to now, it is still unclear whether or not FC could be used as a biomarker for the diagnosis of PI patients at the individual level.

In the present study, we explored whether or not three commonly used FC methods (i.e., voxel-wise FCS, large-scale FC and ReHo; please see the next section for details) could be used as potential biomarkers for the classification of individual patients with PI. This was performed using multivariate pattern analysis (MVPA) with linear support vector machine (SVM) (20).

## Methods

### Participants

This retrospective study was approved by the ethics committee of Guangdong Second Provincial General Hospital and all participants provided written informed consent after they were provided a complete description of the study. Thirty-eight patients with PI (16 men; mean ± standard deviation age, 40.61 years ± 9.43) were recruited from the Guangdong Second Provincial General Hospital.

A total of 38 subjects with PI were recruited. The inclusion criteria for PI patients were: (a) all patients must meet the Diagnostic and Statistical Manual of Mental Disorders, Fourth Edition (DSM-IV) for diagnosis of PI; (b) patients complained of difficulty falling asleep, maintaining sleep, or early awakening from sleep for at least 1 month; (c) patients had no other sleep disorders such as hypersomnia, parasomnia, sleep-related movement disorders, or other psychiatric disorders; (d) patients were younger than 60 years old (e) free from any psychoactive medication for at least 2 weeks prior to and during the study; (f) patients were right-hand dominant as assessed using the Edinburgh Handedness Inventory. Exclusion criteria were as follows: (a) patients had an abnormal signal in any region of the brain which was verified by conventional T1-weighted or T2-fluid-attenuated inversion recovery MR imaging; (b) the insomnia disorder was caused by organic disease or severe mental disease that was secondary to depression or generalized anxiety; (c) other sleep disorders; (d) women who were pregnant, nursing, or menstruating. Although all the subjects were collected using the DSM-IV criterion, the DSM-V has been published. According to DSM-V, participants must have substantial distress or daytime impairment per week that lasts for at least 3 month (1 month for DSM-IV) to be diagnosed with insomnia. Therefore, we further chose those with a duration of more than 3 months. Most future studies may adopt the DSM-V criteria, so we tried to select the subjects who meet the DSM-V criteria as far as possible in order that future studies can reproduce our research. A total of 44 age-, gender-, and education-matched healthy control subjects were recruited (11 men and 33 women; age, 39.91 years ± 9.43) from the local community by advertisements. HC met the following criterion: (a) Insomnia Severity Index (ISI) score <7; (b) no history of swing shifts, shift work, or sleep complaints; (c) no medication or substance abuse such as caffeine, nicotine, or alcohol for at least 2 weeks prior to and during the study; (d) no brain lesions or prior substantial head trauma, which was verified by conventional T1-weighted or T2-fluid-attenuated inversion recovery MR imaging; (e) no history of psychiatric or neurological diseases; (f) right-hand dominant. All the patients were part of previous studies (21–23). All previous studies were investigations of between-group differences using resting-state functional MR imaging, whereas the present study explored whether resting-state functional MR imaging could be used as a neuroimaging biomarker to identify primary insomnia.

Several questionnaires were completed by the study participants. These questionnaires included the ISI, the Pittsburgh Sleep Quality Index (PSQI), the Self-rating Anxiety Scale (SAS), and the Self-rating Depression Scale (SDS).

### Image Acquisition

Functional MR imaging was acquired using a 1.5 Tesla MR scanner (Achieva Nova-Dual; Philips, Best, the Netherlands) in the Department of Medical Imaging, Guangdong Second Provincial General Hospital. Participants were instructed to rest with their eyes closed and remain still without falling asleep. Functional MR images were acquired in about 10 min using a gradient-echo planar imaging sequence as follows: interleaved scanning, repetition time/echo time = 2,500 ms/50 ms, section thickness = 4 mm, intersection gap = 0.8 mm, matrix = 64 × 64, field of view = 224 mm × 224 mm, flip angle = 90°, 27 axial slices, and 240 volumes. After the scan, all subjects were asked if they were asleep during the scan. Those subjects who fallen asleep were excluded.

### Data Preprocessing

Functional images were preprocessed using the SPM12 software package and the Data Processing Assistant for Resting-State fMRI software (DPARSF, Advanced Edition, V4.3) (http://www.rfmri.org/DPARSF) (24). The first 10 images of each participant were discarded to allow the signal to reach equilibrium. Subsequently, the resting-state fMRI data was corrected for temporal differences between slices and head motion. All participants had no more than 2.0 mm of maximal displacement and 2.0 of maximal rotation in any direction. Next, the corrected fMRI data were spatially normalized to the standard Montreal Neurological Institute (MNI) template and were resampled to 3 × 3 × 3 mm^3^. We further processed the data to remove linear trends and filtered temporally (band-pass, 0.01–0.1 Hz). Finally, nuisance signals, including 24 head motion parameters, CSF signals, white-matter signal, and global signal were regressed out from the fMRI data.

### Whole-Brain Voxel-Wise FCS Analysis

Whole-brain voxel-wise FCS as well as large-scale FC (large-scale FC) and ReHo analysis were performed using DPARSF (http://www.rfmri.org/DPARSF). For each participant, all voxels' time series were extracted, and then the Pearson's correlation coefficients between the time series of all pairs of voxels were obtained to form a whole-brain voxel-wise FC matrix. Then, for each voxel, a FCS value was calculated as the sum of the Pearson's correlation coefficients between each voxel and all other voxels. According to previous studies (25–27), we set a threshold of *r* = 0.25 to remove weak correlations possibly arising from signal noise as well as negative correlations. Consequently, we obtained a 3D FCS map for each participant. Finally, the FCS map was converted to *z* scores using Fisher transformation and further spatially smoothed with a 6 mm full-width at half maximum isotropic Gaussian kernel. It is worth noting that this computation was constrained within a gray matter mask, which was created by setting a threshold of 0.2 on the SPM12's gray matter probability template.

### Whole-Brain Large-Scale FC Analysis

Nodes were demarcated by a 268-node functional atlas (28), which was defined using a group-wise spectral clustering algorithm (29), and consequent analysis were similar to previous studies (30). The time series for each node was extracted for each participant by averaging the time series throughout all voxels for each node. FC between each pair of nodes was calculated using Pearson's correlation analysis, which produced (268 × 267)/2 = 35,778 dimensional FC feature vector for each participant. Finally, Fisher transformation was performed for FC.

### Whole-Brain ReHo Analysis

ReHo calculation was also constrained within the same gray matter mask similar to the whole-brain voxel-wise FC analysis. For each voxel of each participant, a ReHo value was calculated by calculating Kendall's Coefficient of Concordance (KCC) of the time series for the given voxel with those of its 26 neighbors (31). A 3D ReHo map was obtained for each participant. We further normalized the ReHo map by dividing the ReHo value for each voxel by the averaged ReHo value of the whole brain. Finally, all ReHo maps were smoothed using a 6 mm full-width at half maximum isotropic Gaussian kernel.

### Multivariate Pattern Classification Analysis

**Figure 1:**
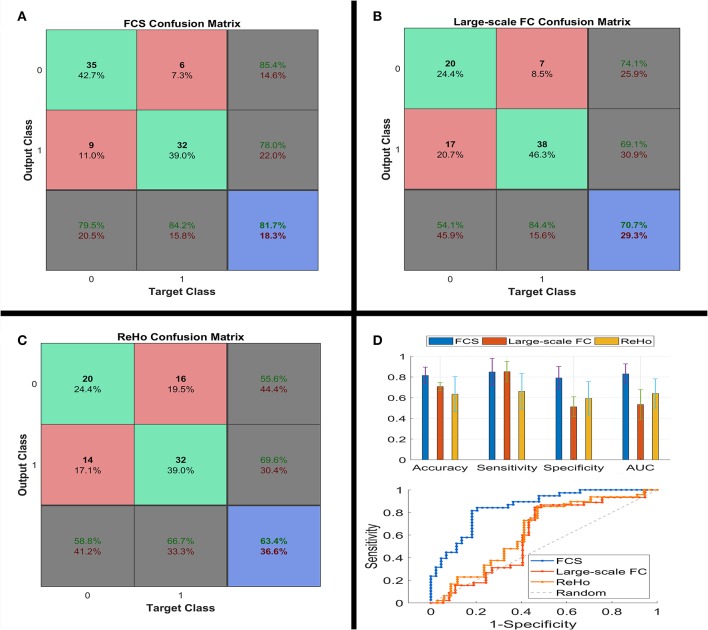
Confusion matrix **(A–C)**, classification performances (the upper part of **D**) and ROC (the lower part of **D**) of linear SVM classifier using the three types of the functional connectivity features. ROC, receiver operating characteristic; SVM, support vector machine.

The MATLAB codes used in our analysis are available online: https://github.com/lichao312214129/lc_rsfmri_tools_matlab/tree/master/Machine_Learning/Classification (SVM_LC_Kfold_PCA _*.m). Our analysis consisted of a 5-fold cross-validation procedure for each of the 3 metrics (i.e., FCS, large-scale FC and ReHo). At each fold *k* (*k* = 1, 2, 3, 4, 5), data of both PI and HC were divided into 2 subsets of 8 to 2. Then the 2 larger subsets from both groups were fused together to form the training data (80%), with the others being test subsets (20%) and only used to assess generalization performance. Normalization and principal component analysis (32) were further performed on the training data. Testing data was also processed by these 2 processes using the same parameters (e.g., principal component coefficients) from the training data. We retained all the principal components, so the principal component analysis just amounts to a coordinate transformation (32). Then, a linear SVM classifier was trained on the training data and used to classify the testing data. By comparing the predicted labels with the real labels, we acquired the classification performances [i.e., accuracy, sensitivity, specificity, and area under ROC curve (AUC)] of one fold. Moreover, discriminative weights were obtained as linear SVM weights (i.e., Beta values of features from the linear SVM classifier). Final classification performances and discriminative maps were acquired as the average over the 5 folds. At the end of the iteration, we acquired the prediction labels for every participant, which was used to build the confusion matrix (please see Figures 1A–C).

### Statistical Analysis

Demographic and scale data of all participants were analyzed using SPSS (version 20; SPSS, Chicago, III). Differences in age, education level, ISI, PSQI, SAS, and Self-rating Depression Scale scores (SDS) between PI patients and healthy controls were compared using Wilcoxon rank sum tests. Differences associated with age were assessed using chi-squared tests.

Non-parametric permutation testing was used to estimate the statistical significance of the averaged classification performances by determining whether these performances exceeded chance levels. The class labels (i.e., PI patients vs. HC) of the training data were randomly permuted 1,000 times prior to training, and repeated the entire 5-fold cross-validation procedure. The *P* value of the permutation test was defined as: *P* = (N_exceed_+1)/ (N_permutation_+1). Where N_exceed_ represents the number of times the permuted performance exceeded the one obtained for the true labels. N_permutation_ represents the times of permutation.

Because of the unfavorable classification performance of the large-scale FC and ReHo (please see Figure 1), we only performed the permutation test on FCS. We additionally analyzed the between-group differences of these three FC metrics using traditional two-sample *t*-test, with age, sex and years of education as covariates. Since the focus of this study is FCS, correlation analysis was conducted to determine whether FCS was correlated with clinical characteristics in the PI group.

## Results

### Demographic and Scale Data

As shown in Table 1, the PI patients and the controls showed no significant differences in age (*P* = 0.74), sex (*P* = 0.10), and education level (*P* = 0.19). However, PI patients had higher ISI, PSQI, SAS, and SDS scores compared to HC (all *P* < 0.001).

**Table 1:** Demographic and scale data of all study participants.

**Variables**	**PI group (*n* = 38)**	**HC group (*n* = 44)**	***P* value**
Gender (M/F)	16/22	11/33	0.10^*^
Age (y)	40.61 ± 9.43	39.91 ± 9.43	0.74^#^
Duration (mo)	40.31 ± 40.09	N/A	N/A
Education (y)	7.50 ± 3.54	8.45 ± 4.31	0.19^#^
ISI	19.32 ± 3.09	5.43 ± 2.46	< 0.001^#^
PSQI	12.45 ± 3.09	5.77 ±3.15	< 0.001^#^
SAS	50.29 ± 11.29	39.73 ± 5.68	< 0.001^#^
SDS	55.21 ± 9.57	40.39 ± 2.54	< 0.001^#^

*Unless otherwise noted, data are mean ± standard deviation*.

* *The P value was obtained using the chi-square test*.

# *The P values were obtained using the Wilcoxon rank sum tests*.

*N/A, Not Available; PI, Primary Insomnia; HC, Healthy Control; ISI, Insomnia Severity Index; PSQI, Pittsburgh Sleep Quality Index; SAS, Self-rating Anxiety Scale; SDS, Self-rating Depression Scale*.

### Classification Performances

Figure 1 shows the confusion matrix and classification performances of the 3 metrics. FCS reached 81.5 ± 9.0% for accuracy, 84.9 ± 14.7% for sensitivity, 79.1 ± 12.3% for specificity, and 83.0 ± 10.8% for AUC (all *P* < 0.001). However, several performances for large-scale FC and ReHo were around 50%, i.e., the chance level. Consequently, the focus of our study was only on FCS.

### Classification Weight Maps

**Figure 2:**
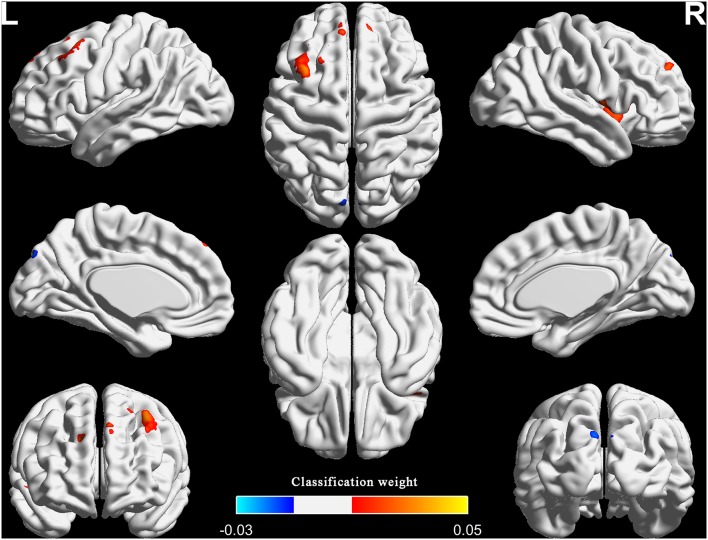
The top one percent of classification weight maps from the linear SVM classifier using the FCS as feature (cluster size threshold = 100). FCS, functional connectivity strength.

Figure 2, Table 2, and Figure S1 show the top one percent of classification weight maps from linear SVM classifier using the FCS (cluster size threshold = 100). Right anterior insular cortex, left precuneus and left middle frontal gyrus contributed high weight to the classifier.

**Table 2:** Top one percent of the region weights from SVM classifier using the functional connectivity strength.

**Brain regions**	**MNI coordinates (Peak)**			**Cluster size (Voxels)**	**Weight (Peak)**
	**X**	**Y**	**Z**		
Anterior insular cortex (R; BA48)	39	3	−6	122	0.03
Precuneus (L; BA7)	−3	−66	60	114	−0.03
Middle frontal gyrus (L; BA8)	−33	21	51	212	0.05

*L, Left; R, Right*.

### MVPA Results of More Rigorous Inclusion Criteria

Considering that previous research used more rigorous inclusion criteria: duration > 6 month, total sleep time ≤ 6.5 h and either sleep onset latency (SOL) > 45 min or WASO > 45 min or SOL + WASO > 60 min (33, 34), we also adopted the additional specific severity criteria to the patients group and repeated the MVPA for FCS (number of patients = 22; duration = 61.7 ± 69.2; total sleep time = 326.8 ± 35.0; SOL = 46.8 ± 2 8.8; WASO = 95.0 ± 52.3). Results showed that the classification performances were 76.6 ± 9.3% for accuracy, 76.3 ± 9.5% for sensitivity, 76.9 ± 13.7% for specificity, and 86.0 ± 7.0% for AUC. The right anterior insular cortex, left middle frontal gyrus and bilateral superior frontal gyrus had relatively high classification weights (right anterior insular cortex and left middle frontal gyrus were the repeated regions in the two analyses). We reported the results that adopted the specific criteria in the Figure S2.

In addition, as to the healthy controls, substantial studies reported that PSQI total score < 5 was defined to the healthy controls (35–37). In order to minimize the influence of PSQI on the results, we use linear regression method to remove the covariate PSQI. Then, we repeated the MVPA for FCS. Results showed that the classification performances were 82.9 ± 5.9% for accuracy, 84.7 ± 13.4% for sensitivity, 80.7 ± 13.1% for specificity, and 90.7 ± 5.1% for AUC. The right anterior insular cortex and left middle frontal gyrus had relatively high classification weights (these two regions were all the repeated regions in the two analyses). We reported the results in the Figure S3.

### Between-Group Differences and Correlation Analysis

**Figure 3:**
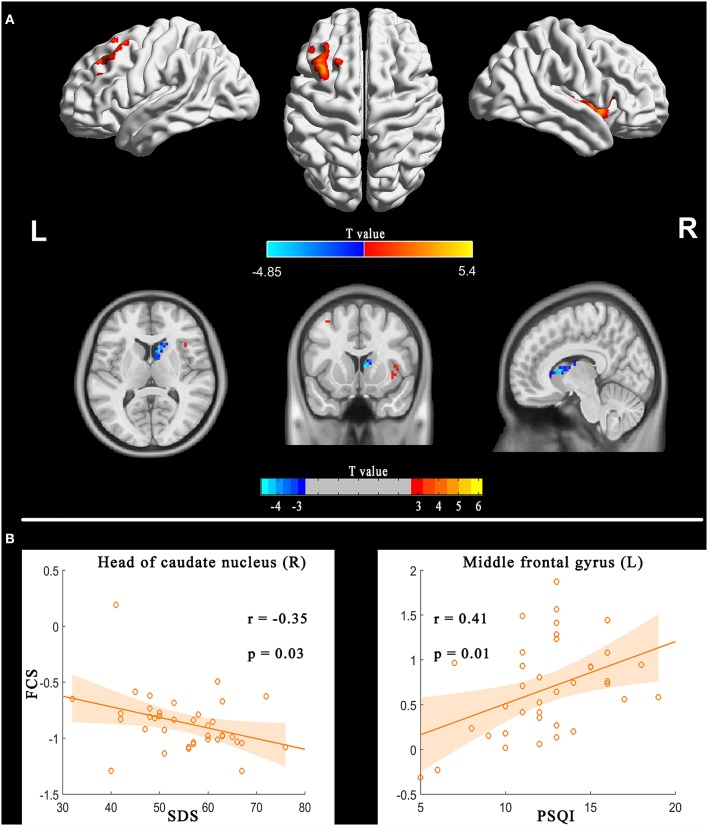
FCS differences between PI patients and HC (PI-HC, **A**) and correlation between FCS and sleep scales in the PI group **(B)**. The threshold was *P* < 0.01 at the voxel level, with Alphasim corrections for multiple comparisons of *P* < 0.05. The color bar represents the *t* value. FCS, functional connectivity strength; PI, primary insomnia; HC, healthy controls.

Figure 3, Figure S4, and Table 3 illustrate the regions showing between-group differences in FCS maps (Alphasim correction for multiple comparisons of *P* < 0.05 combined with single voxel *P* < 0.01). The estimated Gaussian filter widths (FWHM, in mm) were [7.161, 7.834, and 7.771]. The number of Monte Carlo simulations was 1000. Compared with HC, PI patients showed increased FCS in right anterior insular cortex and left middle frontal gyrus, while decreased in the right head of the caudate nucleus. It is worth noting that the right anterior insular cortex and left middle frontal gyrus also showed high classification weights.

**Table 3:** Between-group differences (PI-HC) for functional connectivity strength.

**Brain regions**	**MNI coordinates (Peak)**			**Cluster size (Voxels)**	***T* values (Peak)**
	**X**	**Y**	**Z**		
Anterior insular cortex (R; BA48)	36	3	−6	100	5.40
Head of caudate nucleus (R; BA25)	9	12	9	70	−4.85
Middle frontal gyrus (L; BA8)	−33	21	51	64	4.58

*L, Left; R, Right. The significance level was set to P < 0.01 at the voxel level, with Alphasim corrections for multiple comparisons of P < 0.05*.

**Figure 4:**
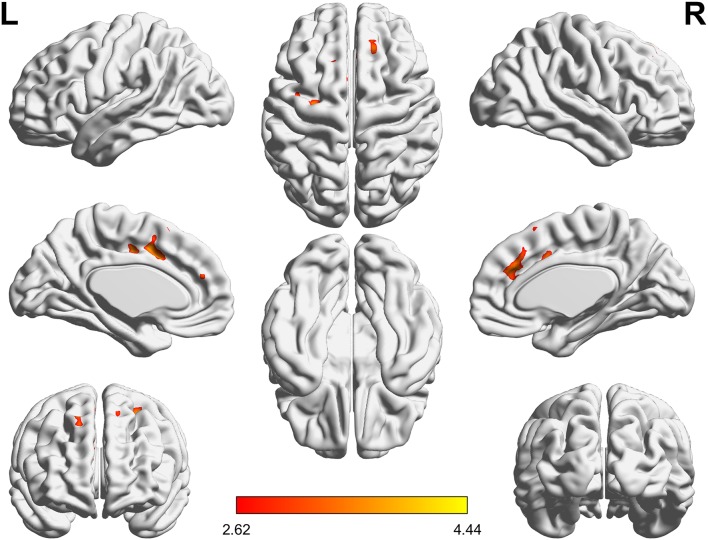
ReHo differences between PI patients and HC (PI-HC). The threshold was *P* < 0.01 at the voxel level, with Alphasim corrections for multiple comparisons of *P* < 0.05. The color bar represents the *t* value. ReHo, regional homogeneity; PI, primary insomnia; HC, healthy controls.

In addition, we found that patients with primary insomnia showed increased ReHo in bilateral anterior cingulate gyrus, left precentral gyrus and superior frontal gyrus (Alphasim correction for multiple comparisons of *P* < 0.05 combined with single voxel *P* < 0.01; Figure 4). The estimated Gaussian filter width (FWHM, in mm) were [7.276, 8.014, and 7.841]. The number of Monte Carlo simulations was 1000. However, larger scale FC showed no between-group difference (FDR *q* < 0.05).

Correlation analyses showed the FCS in the left middle frontal gyrus and right head of the caudate nucleus were correlated with PSQI and SDS respectively (Figure 3B).

## Discussion

To our knowledge, this is the first study to employ MVPA for the automatic classification of patients with PI using three types of FC features. In the current study, we investigated whether or not the three types of FC metrics could be used as biomarkers to define PI. Specifically, the classification performances of FCS were all approximately equal to or more than 80% for diagnosing PI patients. The right anterior insular cortex (BA48) and left middle frontal gyrus (BA8) not only had higher classification weights, but also were the repeated regions with those of between-group comparison. In addition, correlation analysis showed that FCS in left middle frontal gyrus and head of right caudate nucleus were correlated with PSQI and SDS, respectively.

Convergent findings based on functional MR imaging support that spontaneous neural activity or FC in the insular cortex, prefrontal cortex and precuneus were disrupted in patients with insomnia or subjects with insomnia symptoms (8–14). Here we show that these regions can be used to discriminate patients with PI from HC.

Intriguingly, the right anterior insular cortex and the left middle frontal gyrus not only had high classification weights, but also showed differences between groups. Right anterior insular cortex is a key node of the salience network, and is implicated in arousal and insomnia (9, 38). Chen et al. demonstrated that the anterior insular cortex had greater involvement with the salience network, which indicated that the region was involved in hyperarousal in insomnia, and may be an important target for novel therapies for PI (9). Here we showed that the right anterior insular cortex had increased FCS and can be used to discriminate patients with PI from HC.

The finding regarding anterior insular cortex was right lateralized. Previous study demonstrated that the right anterior insular cortex plays a critical and causal role in switching between the central-executive network and default-mode network for better performance of cognitively demanding tasks, but not the left anterior insular cortex (38). The abnormalities in these networks and abnormal cognitive function were a common observation in studies of insomnia. Therefore, our study further highlighted the importance of the right anterior insular cortex in the neurophysiologic of insomnia. However, our findings that increased FCS in the left middle frontal gyrus were not consistent with previous studies. Reduced metabolism, activation or spontaneous neural activities in the prefrontal cortex are the general findings (6, 12, 22, 39). One explanation might be that increased FCS, a manifestation of increased interaction between a given region and other regions, was compensatory to the above reduction in the prefrontal cortex. Future research needs to verify this hypothesis.

Previous study demonstrated that there are prominent beta and theta oscillations in the middle frontal gyrus during REM sleep and suggested this area may play a role in the regulation of memory consolidation (40). Besides, the middle frontal gyrus belongs to the dorsolateral prefrontal cortex which is thought to be involved in alertness, attention, and higher-order cognitive processes, and all these function are disrupted in patients with insomnia (41). Therefore, dysfunction in the middle frontal gyrus may also related with abnormal memory consolidation and compromised cognitive function in PI patients.

In addition, we also found decreased FCS in the head of the caudate nucleus, which also negatively correlated with SDS. Previous studies have established that the caudate is involved in the most consistently reported abnormalities for insomnia, i.e., hyper-arousal, sleep problems and deficits in working memory, episodic memory, and problem solving (15). Furthermore, stimulating the caudate could reduce excitability of the human cortex (42). Using functional MR imaging, Stoffers et al. found that hyper-arousal, a most prominent characteristic of insomnia, was associated with reduced caudate recruitment when performing an executive task (15). Interestingly, our study found that the functional interaction between the head of the caudate nucleus and other brain regions was weaker at the resting state. Considering that caudate nucleus can inhibit brain excitation, decreased FCS in this area may reflect a decreased inhibition of the caudate nucleus on brain cortex. Although FCS in the head of the caudate nucleus cannot be used to identify insomnia, decreased FCS in this region might be the underlying neurobiological substrate for hyper-arousal in insomnia.

Besides, we also found increased ReHo in bilateral anterior cingulate gyrus. This finding is in line with previous study (43). Another previous study using PET-CT also found that patients with PI have high glucose metabolism in the anterior cingulate gyrus compared with HC when falling asleep (6). Genes in the anterior cingulate gyrus control circadian rhythms, and are dysregulated in depression (44). In addition, increased functional connectivity in the anterior cingulategyrus is associated with both sleep and depression (45). We speculated that increased ReHo in the anterior cingulate gyrus was correlated with hyperarousal emotional activity in PI patients.

Overall, above mentioned findings were related with hyper-arousal, emotion and cognition. Hyperarousal brain activity and emotional may led to difficulty in falling asleep, then cause cognitive impairment in the day.

Several limitations of the current study have to be acknowledged. First, the statistical correlations were derived with comparisons form questionnaires only (Table S1). Although the diagnosis of PI itself is purely subjective and polysomnography (PSG) is only needed to exclude somatic diagnoses, a whole night sleep study is the most objective and quantitative means of diagnosing and describing PI. Second, a cohort of 38 PI patients and 44 controls is low in order to correctly train an SVM. Additional studies in a large number of PI patients are needed in order to validate the MVPA analysis as a potential biomarker to identify patients with PI. Third, we only used functional MR imaging data. The integration of structural with functional data may be a more effective method to elucidate disease factors that are shared across different metrics. To investigate morphometric changes in brain regions with abnormal FC could be represent a useful approach to better identify the mechanisms underlying the pathogenesis of PI. Forth, we only investigated the static features of the three types of FC and did not study their dynamic features. Increasing evidence has demonstrated that the functional brain connectivity has dynamic characteristics, emergent over time scales spanning milliseconds and tens of minutes. Future studies using dynamic FC are needed when performing MVPA for PI. Fifth, the participants in the present study were all right-hand dominant, therefore, we cannot identify the relation of the R-sided and L-sided findings with handedness. Sixth, concerning the classification performances of FCS, a diagnostic accuracy equal to or more than 80% for diagnosing PI, is a good but not excellent in order to classify PI patients at the individual level (commonly diagnostic accuracy of 100%).

In summary, these limitations notwithstanding, our findings suggest that abnormal FCS in the right anterior insular cortex (BA48) and left middle frontal gyrus (BA8) might serve as a potential neuromarkers for PI.

## Data Availability Statement

The datasets generated for this study are available on request to the corresponding author.

## Ethics Statement

The studies involving human participants were reviewed and approved by Ethics committee of Guangdong Second Provincial General Hospital. Written informed consent to participate in this study was provided by the participants' legal guardian/next of kin.

## Author Contributions

CL, YM, MD, and GJ: guarantors of integrity of entire study and clinical studies. CL, MD, YW, and GJ: literature research. CL, YM, and MD: experimental studies, statistical analysis, and manuscript editing. All authors study concepts, study design, or data acquisition or data analysis/interpretation, manuscript drafting, or manuscript revision for important intellectual content, approval of final version of submitted manuscript, and agrees to ensure any questions related to the work are appropriately resolved.

### Conflict of Interest

The authors declare that the research was conducted in the absence of any commercial or financial relationships that could be construed as a potential conflict of interest.

## Abbreviations

PI: primary insomnia
HC: healthy controls
FC: functional connectivity
Large-scale FC: large-scale functional connectivity
ReHo: regional homogeneity
MVPA: multivariate pattern analysis
SVM: support vector machine
FCS: functional connectivity strength
ROC: receiver operating characteristic curve
AUC: area under the receiver operating characteristic curve
ISI: Insomnia Severity Index
PSQI: Pittsburgh Sleep Quality Index
SAS: Self-rating Anxiety Scale
SDS: Self-rating Depression Scale.
