# Supplementary material for: Multivariate Pattern Classification of Primary Insomnia Using Three Types of Functional Connectivity Features
Source: Front Neurol. 2019 Oct 2;10:1037. doi: 10.3389/fneur.2019.01037 (PMC6783513; doi:10.3389/fneur.2019.01037)
Supplement: Supplementary file 1 [file Table_1.DOCX]

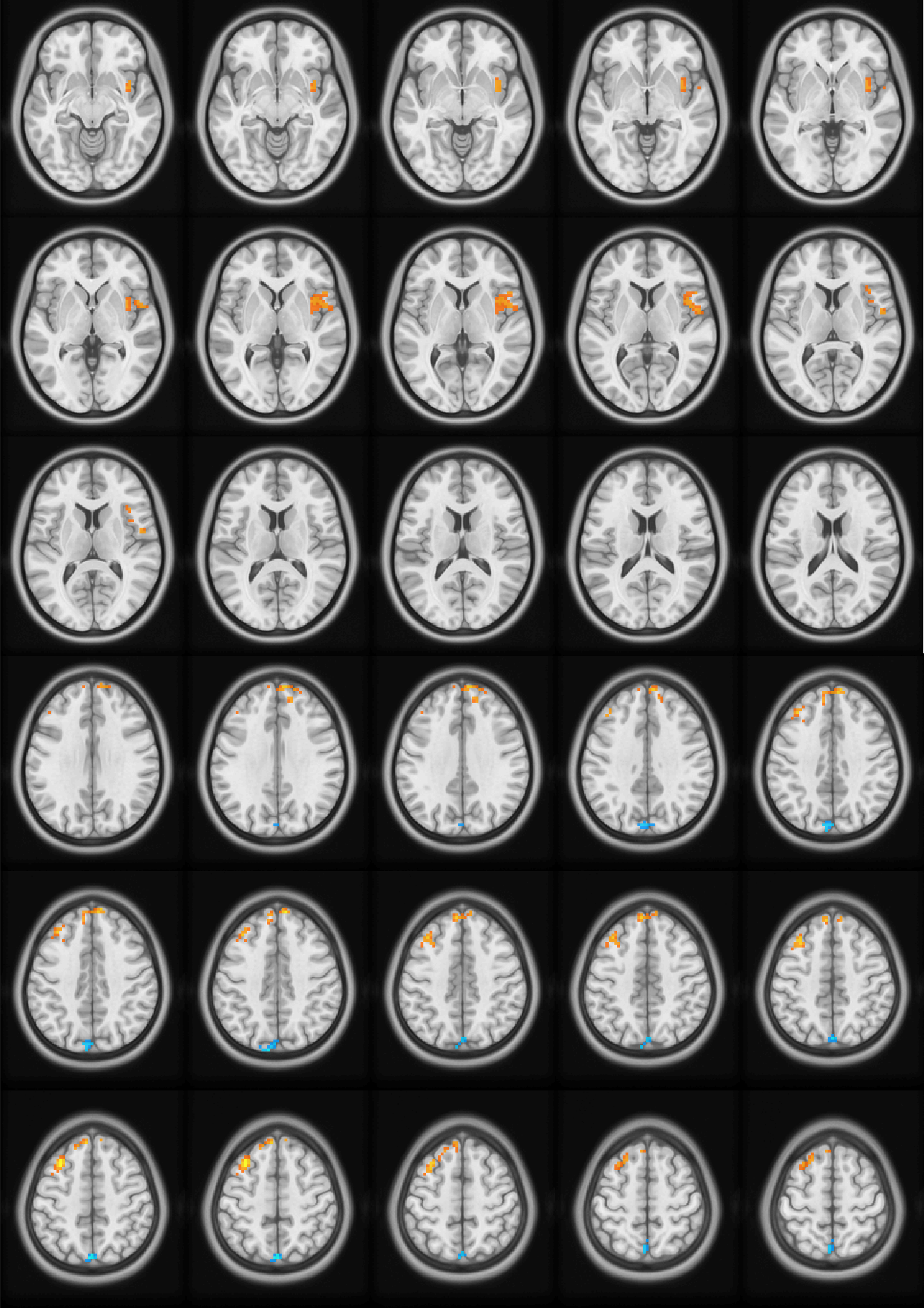


**Figure S1**. The top one percent of classification weight maps from the linear SVM classifier using the FCS as feature (cluster size threshold = 100).

FCS, functional connectivity strength.


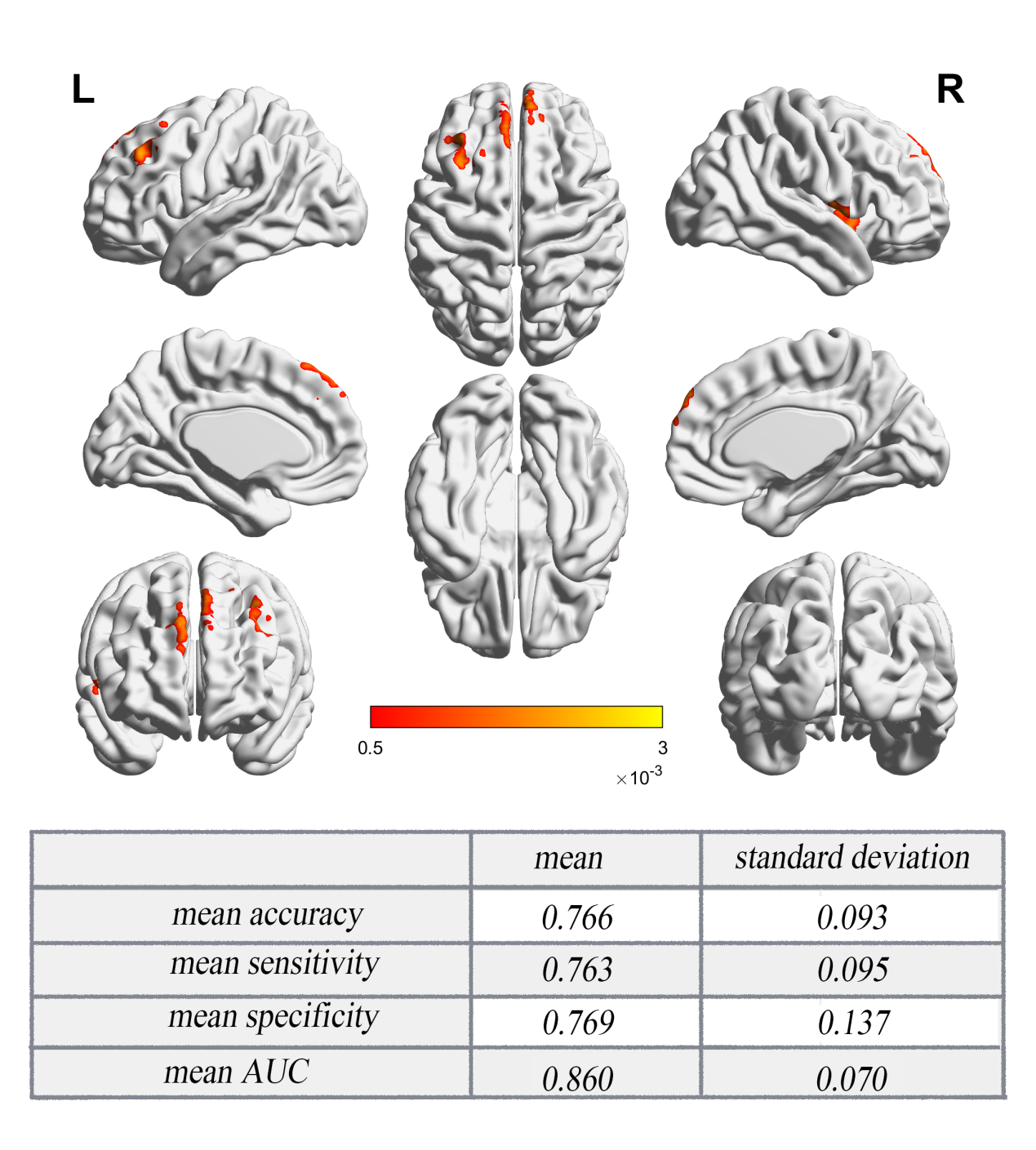


**Figure S2**. The top one percent of classification weight maps from the linear SVM classifier and classification performances using the FCS as feature using more rigorous inclusion criteria (cluster size threshold = 100).

The color bar represents the beta value.

FCS, functional connectivity strength.


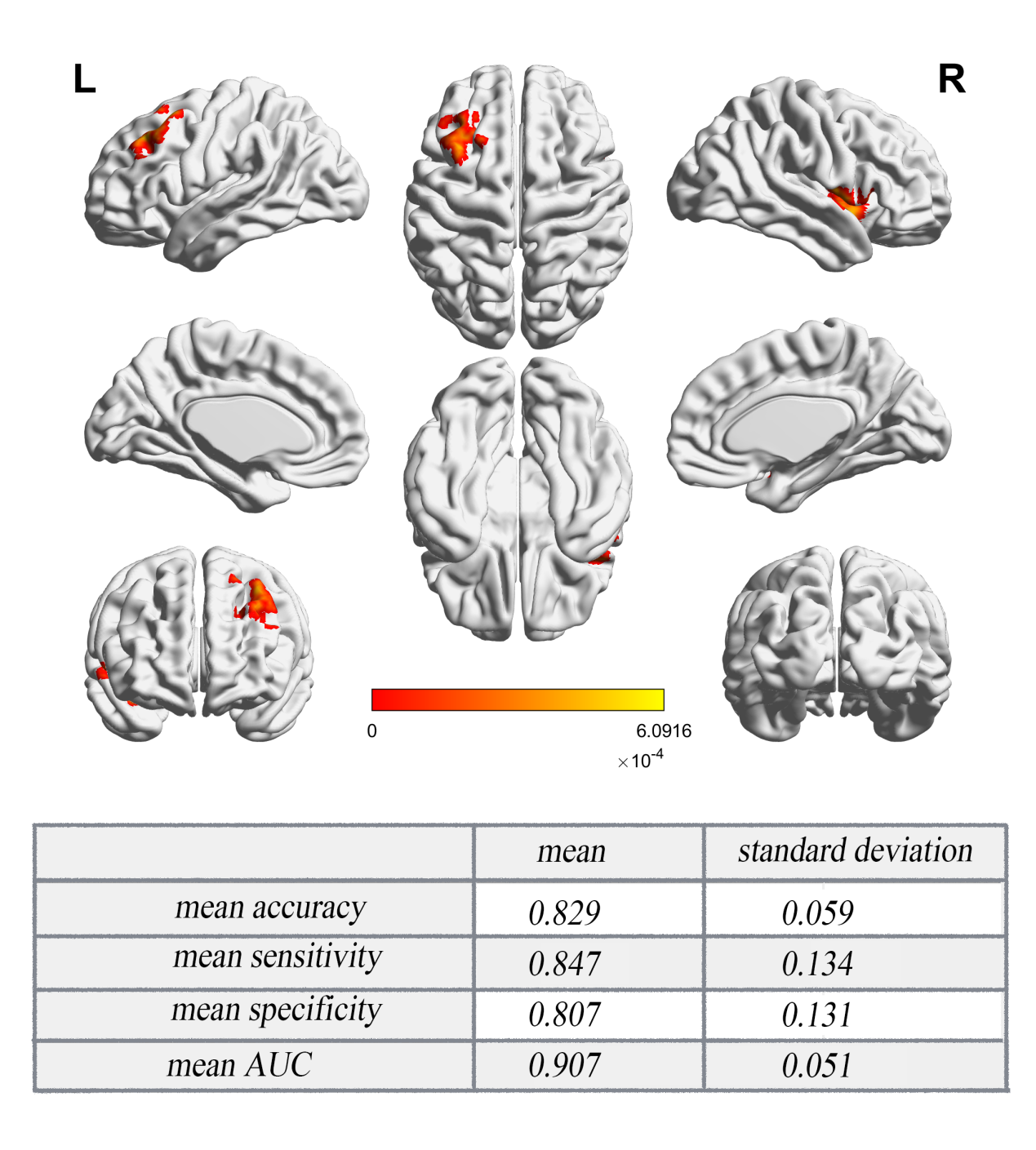


**Figure S3**. The top one percent of classification weight maps from the linear SVM classifier and classification performances using the FCS as feature when control the covariate PSQI (cluster size threshold = 100).

The color bar represents the beta value.

FCS, functional connectivity strength.


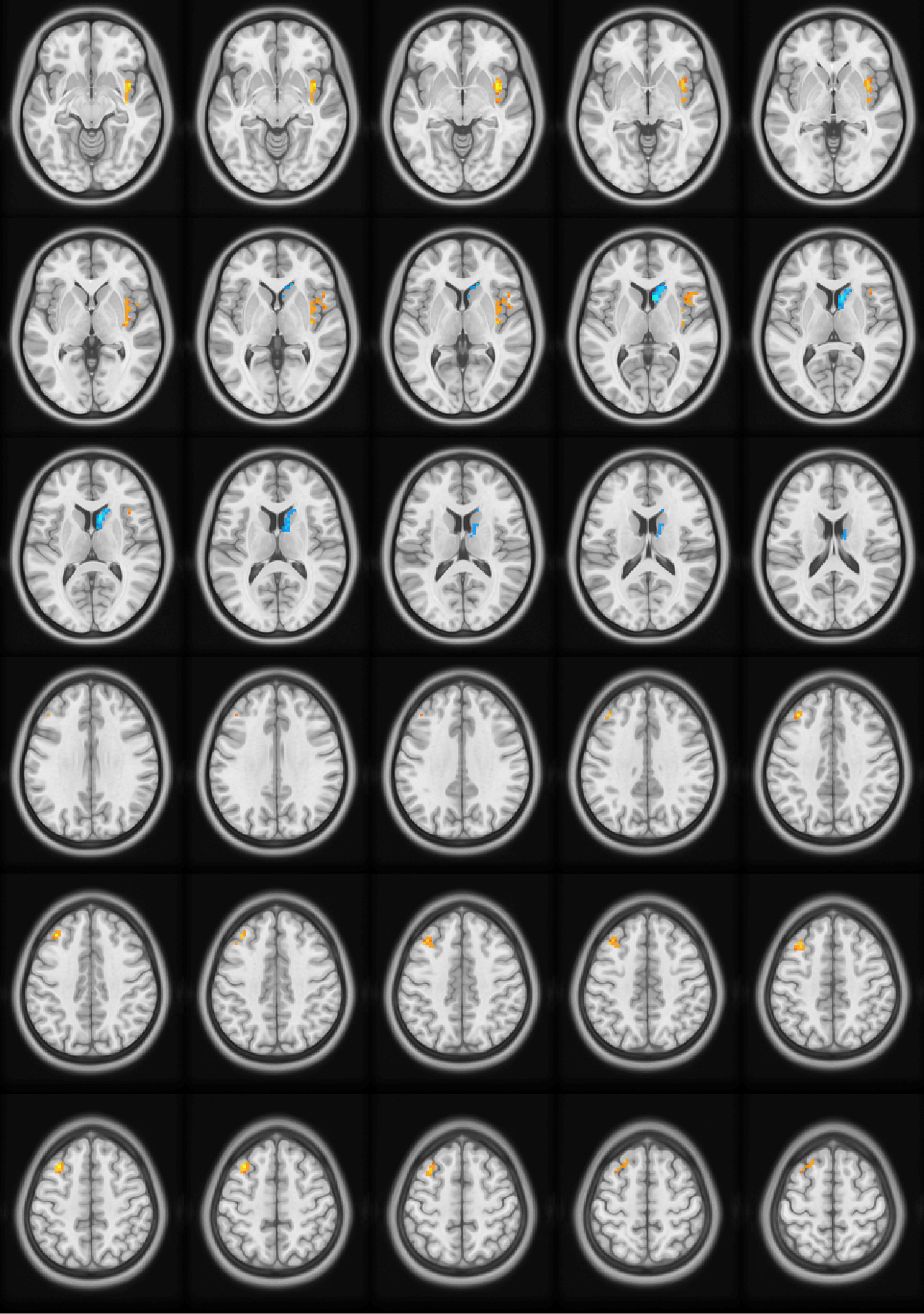


**Figure S4**: FCS differences between PI patients and HC (PI-HC). The threshold was P < 0.01 at the voxel level, with Alphasim corrections for multiple comparisons of P < 0.05.

FCS: functional connectivity strength, PI: primary insomnia, HC: healthy controls.

**Table S1** Subjective sleep parameters in patients with primary insomnia.

SOL TST WASO

count 38 38 38

mean(min) 56.13 348.95 61.97

std(min) 43.07 94.20 66.10

min(min) 3 120 0

max(min) 180 540 270

SOL, sleep onset latency; TST, total sleep time; WASO, wake after sleep onset
